# Do social relationships mediate or moderate social inequalities in health? A systematic review protocol

**DOI:** 10.1186/s13643-022-01973-w

**Published:** 2022-05-14

**Authors:** Nadia Khaliq, Anne McMunn, Carolina Machuca-Vargas, Anja Heilmann

**Affiliations:** 1 Department of Epidemiology and Public Health, UCL, London, UK; 2 Faculty of Dentistry, Oral & Craniofacial Sciences, KCL, London, UK

**Keywords:** Public Health, Social determinants of health, Epidemiology, Social relationships

## Abstract

**Introduction:**

Explanations for health inequalities include material, behavioural and psychosocial pathways. Social relationships are an important determinant of health, and research has consistently found that a lack of support networks may diminish favourable health outcomes. There is some evidence that social network structures, partly shaped by socioeconomic factors, contribute to health inequalities. This protocol will summarise the systematic review process.

**Methods and analyses:**

The Systematic review will be reported according to the Preferred Reporting Items for Systematic Reviews and Meta-Analyses guidelines. An electronic database search of MEDLINE, Embase Classic + Embase and PsychINFO using the OvidSP platform will be undertaken. Databases will be searched from the earliest date of entry until 10 June 2022. Articles that have quantitatively assessed the role of social relationships in mediating or moderating health inequalities will be included and any health outcome (mental/physical) will be considered. The database search will be supplemented by reference list screening of all relevant full-text articles identified through the search. Two independent reviewers will be responsible for screening of articles, data extraction and assessment of bias. Observational studies will be risk assessed for bias using a modified version of the Newcastle-Ottawa Quality Assessment Scale, and intervention studies will be assessed using the revised Cochrane risk-of-bias tool. It is anticipated that the eligible studies will be highly variable; therefore, a meta-analysis will only be considered if the available data of the selected studies are similar. If the studies are too heterogeneous, a narrative synthesis of the extracted data will be presented.

**Conclusion:**

The results of the systematic review will examine the link between social relationships and health inequalities. The findings of the review will identify gaps in knowledge where further research is needed.

**Systematic review registration:**

PROSPERO CRD42020181706

**Supplementary Information:**

The online version contains supplementary material available at 10.1186/s13643-022-01973-w.

## Background

### Social relationships and health

Social inequalities in health are universally recognised as a significant public health problem [1]. The WHO conceptual framework for action on the social determinants of health postulates that structural determinants, including the socioeconomic position of individuals, shape health outcomes through the material, psychosocial and biological factors (intermediate determinants). According to this framework, psychosocial factors include social relationships, with social cohesion and social capital seen as cutting across the structural and intermediary determinants to influence health inequalities [2].

In the UK, tackling health inequalities is an important national strategy. The publication of the White Paper, *‘Healthy Lives, Healthy People’* [3] in response to the Marmot Review [4] led to fundamental legislative change in the government’s approach to tackling the broader structural determinants of health. This change in legislation led to the Health and Social Care Act [5], mandating health bodies, including Public Health England, to reduce health inequalities [6]. However, whilst material and behavioural pathways between socioeconomic position and health have been studied extensively, few studies have explored the contribution of social relationships to social gradients in health.

Social relationships are an umbrella term for the social ties individuals have with others [7] and include aspects such as, but not restricted to, social support, social networks, social capital and social participation. They can be operationalised using either structural or functional social relationship measures and include aspects such as, but not restricted to, social support, social networks, social capital and social participation. Structural social relationship measures refer to the quantitative part of social relationships, for example, marital status, social network size or isolation [8]. Functional measures refer to the qualitative aspect of social relationships, for example, social support and loneliness [8]. Social relationships can also be operationalised at the individual and community level. For example, social support, loneliness and isolation can be viewed as individual level social relationships whereas social participation, social cohesion and social capital are commonly measured at community level. The importance of social relationships for health and well-being is well established [7, 9]. A meta-analysis by Holt-Lunstad et al. [9] concluded that the influence of social relationships on mortality risk is comparable in magnitude to the impact of other well-known risk factors such as obesity and health-compromising behaviours. More unsatisfactory social relationships have also been linked to an increased incidence of coronary heart disease and stroke [10], higher levels of inflammatory markers [11], poorer self-rated oral health and oral-health related quality of life [12], depression [13], cognitive decline [14–16] and altered host susceptibility to disease due to the effect on immune function and neuroendocrine responses [8, 17, 18].

### Evidence of the mediating and moderating role of social relationships in explaining health inequalities

Despite the vast literature on the importance of social relationships for health, evidence regarding the role of social relationships in explaining health inequalities is limited, and results have been contradictory [19, 20].

Evidence suggests potential interactions between markers of socioeconomic position and social relationships and their collective impact on social inequalities in health. Several previous studies assessed whether social relationships can weaken or strengthen the associations between socioeconomic position and health, but results have been highly variable [21–26].

To date, there has been no comprehensive review of the literature on the role of social relationships in mediating or moderating health inequalities. Existing reviews have focused on the role of social capital [27–29]. Carlson and Chamberlain [27] and Vyncke et al. [29] reviewed the role of neighbourhood-level social capital in explaining social inequalities in health. Vyncke et al. [29] limited the population group to children and adolescents, and Carlson and Chamberlain [27] focused on civic trust alone. Similarly, Uphoff et al. [28] only examined whether social capital moderates socioeconomic inequalities in health.

### Research aim

This systematic review aims to assess and synthesise the evidence on the role of social relationships in the association between socioeconomic position and health. The main objectives are:

1. To assess the evidence for the mediating role of social relationships in the association between socioeconomic position and health
2. To assess the evidence for the moderating role of social relationships in the association between socioeconomic position and health

## Methodology

### Protocol and registration

This protocol is registered with PROSPERO (CRD42020181706) and has been reported according to the Preferred Reporting Items for Systematic Reviews and Meta-Analyses Protocols [30] (see Additional file 1).

### Study eligibility criteria

#### Participants

Individuals from any population group and of all ages will be included—there will be no age restrictions or cutoffs for including or excluding studies in this review. Individuals with any physical or mental health condition will also be included.

#### Study design

The review will include any interventional (i.e. randomised or non-randomised trials) or observational study (i.e. cross-sectional, cohort or case-control studies) that assesses the role of social relationships in mediating or moderating health inequalities. Studies need to have quantified the contribution of social relationships to the association between socioeconomic position and health (for mediation) or presented interaction effects or stratified results for different levels of social relationships (for moderation).

Studies will be included if they examine the mediating or moderating role of at least one indicator of social relationships in the association between socioeconomic position and health. The search will include peer-reviewed articles, as well as grey literature (such as preprints and conference abstracts) available from the databases specified below. The only restrictions in terms of publication type will be for review articles, opinion pieces and theoretical articles, which will be excluded.

Studies will be excluded if they only adjusted for socioeconomic position rather than test for the mediating or moderating role of social relationships in explaining health inequalities.

#### Outcomes

An earlier scoping search revealed that there are not many studies that have examined the mediating and moderating role of social relationships in explaining social inequalities in health. As such, this review will include studies that cover a wide range of subjective or objective measures of mental and physical health when assessing health inequalities. Outcome measures will include but will not be limited to, mortality, coronary heart disease, stroke, immune response, self-rated health, health-related quality of life, depression, cognitive decline and measures of oral health. The inclusion and exclusion criteria will not be based on the type of outcome measure used; however, these will be recorded when extracting the data.

#### Exposures

The exposures of interest are socioeconomic position and social relationships. The review will focus on the combined impact of these exposures, i.e. studies will only be included if they explored whether social relationships mediated or moderated the association between socioeconomic position and health.

Socioeconomic position summarises an individual’s position within the social hierarchy and refers to their material and social resources [2]. Studies will not be excluded based on the type of the socioeconomic variable used. Any measure of socioeconomic position will be included such as occupational status, educational level, income, or household wealth; as well as proxy measures such as housing tenure or car ownership, where the indicators mentioned above are not available.

There is no universally agreed-upon definition of what constitutes social relationships; therefore, the search strategy intentionally included terms that refer to the degree of connection individuals have with others. These included, but are not limited to, social connectedness, social support, social networks, engagement, sociability, social attachments, social capital and social integration—keeping the definition of social relationships broad will enable identifying studies that differentiate between structural and functional aspects of social relationships.

Studies will not be excluded based on the type, reliability and validity of the social relationship measure used.

Any article published in any language will be retrieved. No date restrictions will be applied to the search strategy, and databases will be searched from the earliest date of entry until 10th June 2021.

### Search strategy

An electronic search will be carried out to identify all relevant studies from the following databases: MEDLINE, Embase Classic + Embase and PsycINFO.

Search terms include terms relating to social relationships, socioeconomic position, and health inequalities. Specific health outcomes will not be included in the search strategy, as any health outcome will be considered (see Additional file 2 for search terms table).

Various tools such as Boolean operators, truncation and proximity indicators will be used to ensure a comprehensive search that identifies all relevant articles (see Additional file 3). The search strategy was developed by NK and JS, and peer reviewed by JS, KA and DM using the Peer Review of Electronic Search Strategies (PRESS) checklist [31]. Reference lists of all relevant full-text papers will be screened to retrieve additional articles not identified through the database search. The study selection process will be summarised in a PRISMA flowchart [32].

### Study selection

All relevant articles retrieved from the database searches will be stored in the reference manager, Mendeley [33]. Full-text articles will be exported to Rayyan QCRI [34] after removal of duplicates. Two reviewers (NK and CMV) will use Rayyan QCRI [34] to independently (i.e. blind to the other reviewers’ decisions) screen titles and abstracts for eligibility. The UCL findit@UCL linking service will be used to retrieve full texts of relevant articles. Any disagreements regarding the inclusion of papers will be resolved through discussions. Any remaining unresolved disputes following this process will go through a tie-break decision from an external reviewer not involved in the review process (AH).

### Data extraction

A data extraction form will be developed and piloted using a sample of eligible studies. The piloting process will ensure reliability in the interpretation and use of the inclusion criteria. Upon finalisation of the data extraction form, the reviewers (NK and CMV) will extract the data and reasons for exclusion will be listed. Any discrepancies will be resolved through consensus meetings.

The reviewers will extract data regarding key elements of each study, including:

- Citation details such as first author and year of publication
- Study population including country or region, sample size and demographic indicators such as age and sex
- Study design, e.g. cross-sectional, longitudinal
- The follow-up period for longitudinal designs
- Whether the study examined mediation or moderation
- Exposure indicators (socioeconomic position and social relationship variables) and health outcomes assessed
- Statistical methods implemented and main results, e.g. ORs, relative risks.

It is expected that outcome measures will be both continuous—with results presented either as standardised or unstandardised beta coefficients; or categorical—with results presented as odds ratios, relative risks or hazard ratios.

### Synthesis

It is anticipated that the eligible studies to be included in the review will be highly variable with regards to populations, settings, study design and outcomes. It is further anticipated that results across studies will take various statistical forms—these results will be converted to a common effect metric. If feasible, i.e. where heterogeneity is low to moderate (*I*^*2*^ < 60%), a random-effects meta-analysis will then be used to pool these estimates in a forest plot. Forest plots will be presented separately by study design for studies that assess mediation and similarly for studies that assess moderation. Where information is missing to calculate a common effect metric, additional information will be requested through contacting the authors directly. Subgroup analysis or meta-regression will be carried out to assess sources of clinical and methodological heterogeneity selected a priori: (i) social relationship type, i.e. structural, or functional, (ii) socioeconomic position marker, (iii) health outcome. The Higgins Thompson *I*^*2*^ test will assist in ascertaining whether heterogeneity (clinical, methodological and statistical) exists provided there are at least ten studies included in the meta-analysis. The *I*^*2*^ test statistic can be imprecise for a small number of studies, for example, a meta-analysis with seven or less studies [35]. The *I*^*2*^ statistic will need to be within a recommended threshold, preferably < 60% [36]. A funnel plot will be used to assess for publication bias. The Economic and Social Research Council Methods Programme guidelines [37] will be used to guide the synthesis and will allow identification of any sources of heterogeneity. If the studies are too heterogeneous, i.e. substantial to considerable heterogeneity whereby *I*^*2*^ statistic > 60% [36], a narrative synthesis of the data will be presented by health outcomes and grouped by:

1. Mediation analyses
  - Self-rated health
  - Mortality, chronic disease, and physical function
  - Depression and mental health
2. Moderation analyses
  - Self-rated health
  - Mortality, chronic disease, and physical function
  - Depression and mental health

If a meta-analysis is not feasible, the results will be presented in a narrative synthesis with effect direction plots. Detailed descriptive tables will be presented in a supplementary file. Results from studies will be reported as either positive (if there is evidence of mediation or moderation), null findings (if there is no evidence of mediation or moderation), mixed findings (if there is evidence of mediation or moderation in subgroups only), or negative findings (if there is evidence of social relationships contributing to increased social inequalities in health). Studies will then be further ordered by risk of bias, using colour-coding, i.e. low (green), moderate (yellow) and high bias (red).

#### Risk of bias

Observational studies will be quality assessed using a modified version of the Newcastle-Ottawa Quality Assessment Scale [38]. Assessments of these studies will include representativeness of participants, statistical methods implemented including identification of potential confounders, handling of missing data, attrition in longitudinal studies, how exposures and outcomes were measured and any significant limitations of the study. The revised Cochrane risk-of-bias tool, RoB 2 [39], will be used to quality assess any randomised trials included in the review. Assessments of these studies will address bias in the randomisation process, the selection of results reported, the handling of missing data, and bias due to failure of reporting on deviations from intended interventions. Non-randomised trials will be risk assessed using the Risk Of Bias In Non-randomized Studies of Interventions (ROBINS-I) tool. Assessments of these studies will address confounding, selection bias, information bias and reporting bias [40].

#### Reporting

To allow for transparency and reproducibility of the findings, the methods and results of this systematic review will be reported according to the Preferred Reporting Items for Systematic Reviews and Meta-Analysis (PRISMA) guidelines [32].

#### Timeline for systematic review

It is anticipated that data extraction will start from 31st June 2021 and a draft manuscript will be completed by January 2022.

## Discussion

The quality and quantity of social relationships impact mortality, morbidity and mental health [7, 8]. Public Health England has acknowledged social isolation and social inequality as being related to poor health outcomes [41]. Therefore, this systematic review will examine the link between social relationships and health inequalities. The indirect link between socioeconomic position and health via social relationships will be investigated with attention being paid to the mechanisms identified—whether social relationships mediate or moderate the association between socioeconomic position and health. The strengths and limitations of the evidence will be considered, and findings will be discussed in context with related reviews. The results of the review will summarise the existing evidence of the impact of social relationships on health inequalities and identify gaps in knowledge where further research is required. It is expected that the findings will be useful for the development of policies aiming to reduce social inequalities in health.

## Supplementary Information

**Additional file 1.** Research Checklist.

**Additional file 2: Table 1.** Search terms.

**Additional file 3.** Tools and techniques for search strategy.

## Data Availability

Data sharing is not applicable to this article as no datasets were generated or analysed during the current study.

## Abbreviations

WHO: World Health Organisation
PROSPERO: International prospective register of systematic reviews
PRISMA: Preferred Reporting Items for Systematic Reviews and Meta-Analyses
