# Supplementary material for: Do social relationships mediate or moderate social inequalities in health? A systematic review protocol
Source: Syst Rev. 2022 May 14;11:91. doi: 10.1186/s13643-022-01973-w (PMC9107128; doi:10.1186/s13643-022-01973-w)
Supplement: Supplementary file 2 — Additional file 2: Table 1. Search terms. [file 13643_2022_1973_MOESM2_ESM.pdf]

Table 1 Search terms

SEARCH TERMS	
**Database**	**MeSH terms**
Medline	Socioeconomic Position – socioeconomic factors/ or economic status/ or poverty/ or poverty areas/ or social class/ or educational status/ or employment/ or income/ or occupations/ or career mobility/ or poverty/ or social conditions/
	Social Relationships – interpersonal relations/ or intergenerational relations/ or family relations/ or social capital/ or social environment/ or community networks/ or social support/ or psychosocial support systems/ or social isolation/ or loneliness/ or social alienation/ or social marginalization/ or friendship/
	Health Inequalities – Health Status/ or Health Status Disparities/
Embase Classic + Embase	Socioeconomic Position – socioeconomic factors/ or economic status/ or poverty/ or poverty areas/ or social class/ or educational status/ or employment/ or income/ or occupations/ or career mobility/ or poverty/ or social class/ or social conditions/
	Social Relationships – interpersonal relations/ or intergenerational relations/ or family relations/ or social capital/ or social environment/ or community networks/ or social support/ or psychosocial support systems/ or social isolation/ or loneliness/ or social alienation/ or friendship/ or social marginalization/
	Health Inequalities – Health Status/ or Health Status Disparities/
PsycINFO	Socioeconomic Position – socioeconomic status/ or educational background/ or educational attainment level/ or parent educational background/ or poverty/ or socioeconomic factors/ or family socioeconomic level/ or socioeconomic factors/ or economic inequality/ or disadvantaged/ or family socioeconomic level/ or income level/ or lower class/ or social class/
	Social Relationships – interpersonal relationships/ or social interaction/ or couples/ or caregivers/ or family relations/ or friendship/ or kinship/ or marital relations/ or partners/ or peers/ or social capital/ or social networks/ or social interaction/ or social inclusion/ or social support/ or support groups/ or social exclusion/ or parasocial interaction/ or social exchange/ or online social networks/ or ingroup outgroup/ or social network analysis/ or social deprivation/ or psychosocial factors/ or social acceptance/ or social groups/ or social isolation/
	Health Inequalities – health inequality.mp./ or health disparities/
**Free-text search terms**	**Medline**

Socioeconomic Position	1. Socioeconomic position MeSH terms
	2. socioeconomic position.mp.
	3. (socioeconomic* or socio-economic* or SES or SEP or income or depriv* or occupation*).ti,ab,kf.
	4. social gradient.mp.
	5. 1 OR 2 OR 3 OR 4
Social Relationships	6. Social relationship MeSH terms
	7. social relationship.mp.
	8. (social relation* or social interaction or social integration or social marginali?ation or social participation or social engagement or social cohesion or social* cohesi*).ti,ab,kf.
	9. 6 OR 7 OR 8
Health Inequalities	10. Health inequalities MeSh terms
	11. health inequality.mp
	12. (health inequ* or health dispar* or health status dispar* or health status dispar* health gradient*).ti,ab,kf.
	13. (health adj2 inequ*).mp.
	14. 10 OR 11 OR 12 OR 13
	15. 5 AND 9 AND 14
	16. Limit to Humans
**Free-text search terms**	**Embase Classic + Embase**
Socioeconomic Position	1. Socioeconomic position MeSH terms
	2. socioeconomic position.mp.
	3. (socioeconomic* or socio-economic* or SEP or SES or depriv* or income or occupation*).ti,ab,kw.
	4. social gradient.mp.
	5. 1 OR 2 OR 3 OR 4
Social Relationships	6. Social relationship MeSH terms
	7. social relationship.mp.
	8. (social relation* or social interaction* or social integration or social cohesion or social engagement or social participation or social marginali?ation).ti,ab,kw.
	9. 5 OR 6 OR 7
Health Inequalities	10. Health inequalities MeSh terms

	11. Health inequality.mp<br>12. (health inequ* or health status dispar* or health dispar* or health gradient*).ti,ab,kw.<br>13. (health adj2 inequ*).mp.<br>14. 10 OR 11 OR 12 OR 13<br>15. 5 AND 9 AND 14<br>16. Limit to Humans
**Free-text search terms**	**PsycINFO**<br>1. Socioeconomic position MeSH terms<br>2. socioeconomic position.mp.<br>3. economic security.mp.<br>4. 1 OR 2 OR 3<br>5. Social relationship MeSH terms<br>6. (social relation*).mp.<br>7. social cohesion.mp.<br>8. 5 OR 6 OR 7<br>9. Health inequalities MeSh terms<br>10. (health inequ* or health dispar* or health gradient).mp.<br>11. health gradient.mp.<br>12. health adj2 inequ*<br>13. 9 OR 10 OR 11 OR 12<br>14. 4 AND 8 AND 13<br>15. Limit to Humans
