# Supplementary material for: Do social relationships mediate or moderate social inequalities in health? A systematic review protocol
Source: Syst Rev. 2022 May 14;11:91. doi: 10.1186/s13643-022-01973-w (PMC9107128; doi:10.1186/s13643-022-01973-w)
Supplement: Supplementary file 3 — Additional file 3. Tools and techniques for search strategy. [file 13643_2022_1973_MOESM3_ESM.pdf]

Supplementary File 3: Tools and techniques for search strategy

Technique	Command	Example
All known synonyms and acronyms of keyword		*socioeconomic position may include socioeconomic status, income, SES, SEP etc.*
Truncation – used to find variant word endings	“root word”*	*depriv* includes deprivation and deprived*
Wildcard – used to replace a character within a word to enable alternative spellings to be included	?	*marginali?ed includes marginalised and marginalised*
Boolean operators – to combine keywords and subject headings within a key concept, and to combine different key concepts	“OR”<br>“AND”	*social position OR social status will identify articles containing either of these terms;*<br>*social capital AND health status disparities will identify articles with both terms*
Searching title, abstract and keyword fields – this will also include current articles to be retrieved that do not yet have MeSH terms assigned	(free-text word search string).ti,ab, kw.	*(health inequ* or health status dispar*).ti,ab.kw.*
